# Clinicopathological features of intraductal papillary mucinous neoplasm derived from ectopic pancreas: A systematic review

**DOI:** 10.1016/j.sopen.2022.03.001

**Published:** 2022-03-15

**Authors:** Jiro Kimura, Takehiro Okabayashi, Kenta Sui, Takahiro Murokawa, Motoyasu Tabuchi, Masaki Aida, Jun Iwata, Yasuhiro Hata

**Affiliations:** a Department of Gastroenterological Surgery, Kochi Health Sciences Center, Kochi City,Japan; b Department of Diagnostic Pathology, Kochi Health Sciences Center, Kochi City,Japan; c Department of Radiology, Kochi Health Sciences Center, Kochi City,Japan

## Abstract

**Background:**

Clinicopathological characteristics of intraductal papillary mucinous neoplasm derived from the ectopic pancreas have not been elucidated owing to its rarity.

**Methods:**

MEDLINE databases from 1985 to 2021 were searched. Data regarding patient characteristics, diagnostic modalities, treatment, and prognosis were extracted from the identified articles.

**Results:**

Comprehensive data on 13 patients (10 men and 3 women) with intraductal papillary mucinous neoplasm derived from ectopic pancreas were extracted. The median age was 69 years (range, 42–80 years). The tumors were located in the stomach in 6 patients, the duodenum in 1 patient, jejunum in 3 patients, ileum in 1 patient, and Meckel diverticulum in 2 patients. Histopathological examination revealed intraductal papillary mucinous neoplasm in 10 patients and intraductal papillary mucinous carcinoma in 3 patients. The median size of the tumor was not significantly different between the intraductal papillary mucinous carcinoma group and the intraductal papillary mucinous neoplasm group (P = .611).

**Conclusion:**

Accurate preoperative diagnosis and differential diagnosis between intraductal papillary mucinous neoplasm and intraductal papillary mucinous carcinoma remain difficult despite recent advances in imaging modalities.

## Highlights

• The ectopic pancreas can be complicated with pancreatitis, pseudocyst formation, cyst formation, insulinoma, adenoma, and malignant transformation. Intraductal papillary mucinous neoplasm (IPMN) derived from the ectopic pancreas is rare and can undergo malignant transformation.
• It is challenging to diagnose IPMN derived from ectopic pancreas preoperatively using the existing imaging modalities. Moreover, the clinicopathological characteristics of these tumors have not been elucidated.
• We performed a review of case reports on IPMNs derived from the ectopic pancreas, including intraductal papillary mucinous carcinoma (IPMC) that was treated in our hospital, to determine the clinicopathological features of the tumors.
• This is the first systematic review of the literature on IPMN derived from the ectopic pancreas. The review revealed that IPMN derived from the ectopic pancreas was diagnosed incidentally in most patients, and some patients were asymptomatic.
• The existing imaging modalities may not accurately diagnose the condition preoperatively and differentiate between IPMN and IPMC. Risk factors for malignant transformation should be clarified through further research.

## INTRODUCTION

The ectopic pancreas is defined as a pancreatic tissue that lacks a direct or vascular connection to the pancreas [1]. However, its origin is unclear. Septation from the main pancreatic structure during embryonic rotation and fusion of dorsal and ventral pancreatic buds is the most supported hypothesis [2]. In an autopsy series, the frequency of ectopic pancreas was reported to range from 0.55% to 13.7% [3]. In a few reported cases, complications caused by ectopic pancreas included pancreatitis, pseudocyst formation, cyst formation, insulinoma, adenoma, and malignant transformation [4,5].

Intraductal papillary mucinous neoplasm (IPMN) is primarily characterized by cystic dilation of the pancreatic duct, with variable degrees of mucin production and epithelial proliferation. To the best of our knowledge, only 12 cases of IPMNs derived from ectopic pancreas have been reported [[6], [7], [8], [9], [10], [11], [12], [13], [14], [15], [16], [17]]. In fact, all previous reports on IPMN derived from ectopic pancreas have been case reports, and no detailed review on this disease exists. In this study, we performed a review of case reports on IPMNs derived from the ectopic pancreas, including intraductal papillary mucinous carcinoma (IPMC) that was treated in our hospital, to determine the clinicopathological features of the tumors.

## PATIENTS AND METHODS

### Literature Search

The current systematic review was carried out according to the Preferred Reporting Items for Systematic Reviews and Meta-Analyses statement [18]. Reports of observational studies written in English or Japanese with abstracts written in English were eligible for inclusion. Only articles for which full-text versions could be retrieved were included.

A literature search was performed using the following terms: *intraductal papillary mucinous neoplasm, ectopic pancreas* and *intraductal papillary mucinous carcinoma, ectopic pancreas*. The PubMed database was searched for articles published between 1985 and 2021, and reports of other studies in the reference lists of the retrieved articles were also included.

### Assessments

Our survey of the literature from 1985 to 2021 revealed 12 patients who had been diagnosed with IPMN derived from the ectopic pancreas, including 1 patient from our institution whose tumor was located in the jejunum [[6], [7], [8], [9], [10], [11], [12], [13], [14], [15], [16], [17]]. All cases were managed in individual departments and described in detail. Clinicopathological data described in these case reports included clinical symptoms, tumor location, type of operation, tumor size, recurrence sites, and survival periods.

### Statistical Analyses

Continuous variables are described as median (minimum to maximum) values. Categorical variables are presented as absolute numbers and percentages. Statistical analyses were performed using Mann–Whitney *U* test for continuous variables and Fisher Exact Test for categorical variables. All statistically significant preoperative and perioperative variables were included to establish the model. Survival rates were generated using the Kaplan–Meier method. All *P* values reported are 2-sided. Statistical analyses were performed using EZR software (Saitama Medical Center, Jichi Medical University, Saitama, Japan).

## RESULTS

### The Patient Who Was Treated in Our Hospital

**Fig 1:**
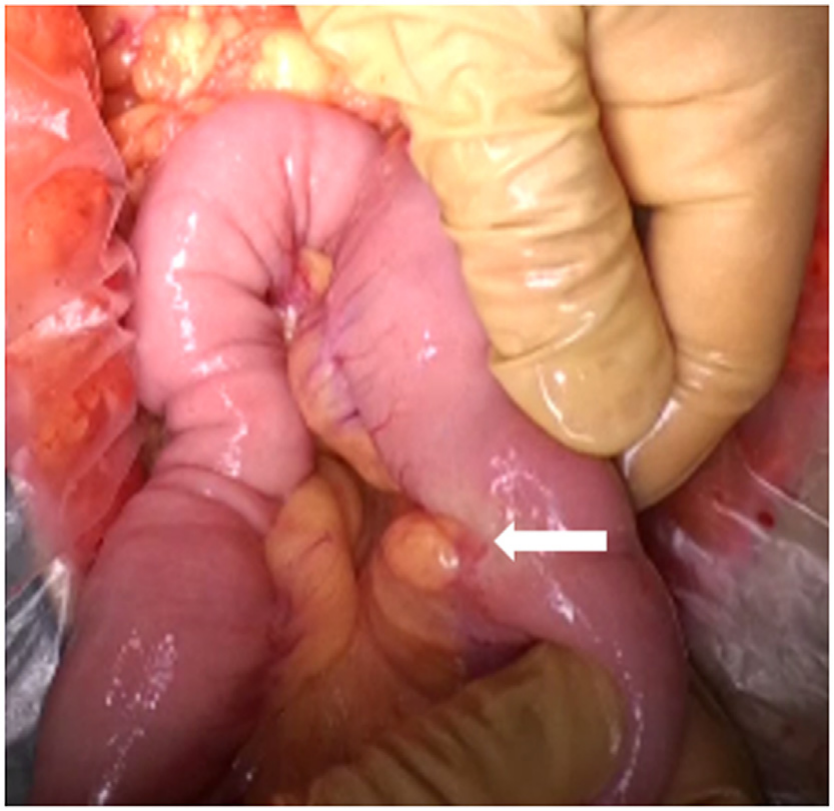
A nodule with a diameter of 2 cm is present in the jejunum, 20 cm from the ligament of Treitz (arrow).

**Fig 2:**
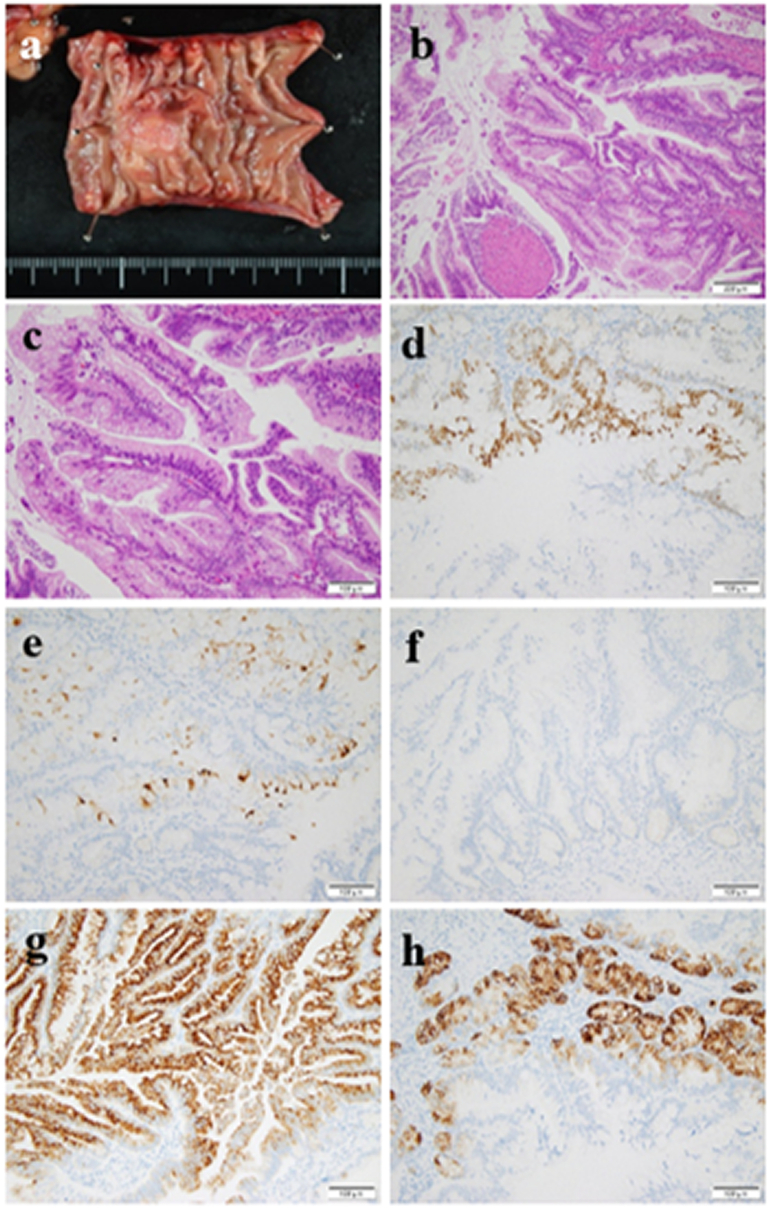
A, Gross observation of the resected specimen reveals a submucosal lesion with a maximum diameter of 18 mm. B and C, Histopathological examination reveals neoplasm with atypical nuclei and tubular structure (B, low-power field; C**,** high-power field). D–H, Immunochemistry confirms the diagnosis of the gastric type of IPMC. D, CDX2(±). E, MUC1(±). F, MUC2(−). G, MUC5AC(+). H, MUC6(+).

A 69-year-old man had presented with epigastric pain to the previous hospital. Contrast-enhanced computed tomography (CT) revealed a pancreatic tumor invading the transverse colon. Biopsy of the tumor in the transverse colon indicated pancreatic adenocarcinoma. He developed acute pancreatitis followed by intra-abdominal abscess formation. Subsequently, he was referred to our hospital for further treatment. Radical surgery was planned after transgastric tube placement into the abscess cavity by upper endoscopy. The tumor was found in the pancreatic tail and had infiltrated the transverse colon and stomach. Therefore, a distal pancreatectomy with partial resection of the transverse colon and stomach was performed. Incidentally, a nodule with a diameter of 2 cm was found in the jejunum, 20 cm from the ligament of Treitz, and subsequently, partial small bowel resection was performed (Fig 1). Histopathological examination of the jejunal nodule revealed IPMC derived from the ectopic pancreas (Fig 2). The patient's postoperative course was uneventful. He is alive with multiple liver metastases clinically suspected from pancreatic cancer, which were observed 3 months after the operation.

### Literature Review

**Fig 3:**
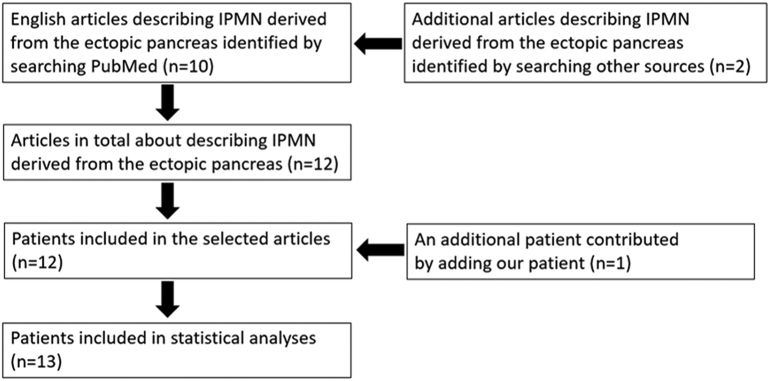
Preferred Reporting Items for Systematic Reviews and Meta-Analyses design showing selection of articles and cases for review.

We identified 10 articles available in electronic databases by searching PubMed and 2 articles from other sources (Fig 3). We extracted comprehensive data from 13 patients with IPMN derived from the ectopic pancreas, including a patient who had been treated in our hospital. The published diagnoses for all patients were true indications for medical management of IPMN derived from the ectopic pancreas.

### Clinical Features of IPMN Derived From Ectopic Pancreas

**Fig 4:**
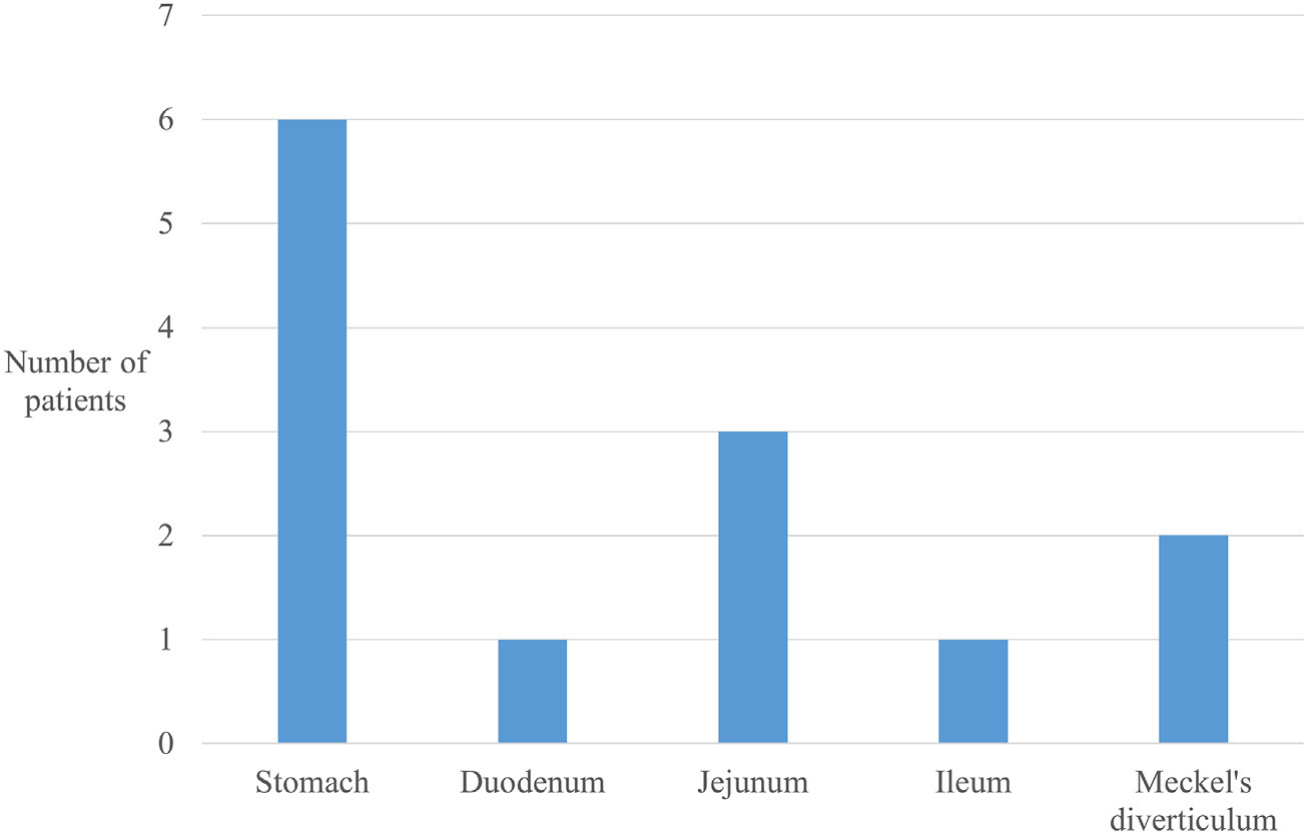
Locations of IPMN derived from the ectopic pancreas.

Of the 13 patients with IPMN derived from the ectopic pancreas, for which we found comprehensive data, 10 were men, and 3 were women. The median age was 69 years (range, 42–80 years). The clinicopathological characteristics of these cases are shown in Table 1. The tumors were located in the stomach in 6 patients, duodenum in 1 patient, jejunum in 3 patients, ileum in 1 patient, and Meckel diverticulum in 2 patients (Fig 4). The most common symptom was abdominal pain in 6 patients. Other symptoms included anorexia, bloating, and diarrhea in 1 patient and jaundice in another patient. The remaining 5 patients had no symptoms. Comorbidities included hypertension in 4 patients, diabetes mellitus in 2 patients, stroke in 2 patients, chronic kidney disease in 1 patient, and malignant disease in 4 patients.

**Table 1:** Clinical characteristics of IPMN derived from ectopic pancreas

*Characteristics*	*Total* N *= 13*
Age (range)	69 (42–80)
Sex (male/female)	10/3
Location of ectopic pancreas	
Stomach	6 (46.2)
Duodenum	1 (7.7)
Jejunum	3 (23.1)
Ileum	1 (7.7)
Meckel diverticulum	2 (15.4)
Symptoms (%)	
Abdominal pain	6 (46.2)
Anorexia	1 (7.7)
Bloating	1 (7.7)
Diarrhea	1 (7.7)
Jaundice	1 (7.7)
None	5 (38.5)
Comorbidities	
Hypertension	4 (30.8)
Diabetes mellitus	2 (15.4)
Stroke	2 (15.4)
Chronic kidney disease	1 (7.7)
Malignant disease	4 (30.8)
Colon cancer	1 (7.7)
Gastric cancer	1 (7.7)
Pancreatic cancer	1 (7.7)
Thyroid cancer	1 (7.7)

### Diagnosis of IPMN Derived From Ectopic Pancreas

Diagnostic modalities for IPMN derived from the ectopic pancreas depended on the location of tumors (Table 2). In the stomach, tumors were detected using esophagogastroduodenoscopy (EGD), including endoscopic ultrasound in 2 patients, magnetic resonance imaging (MRI) in 2 patients, CT in 1 patient, and incidentally during operation in 1 patient. The tumors in the duodenum and ileum were diagnosed using CT. In the jejunum, the tumors were incidentally detected during surgery for abdominal aortic aneurysm in 1 patient and pancreatic cancer in another patient. Remarkably, in 2 patients, the tumors were detected in the Meckel diverticulum during exploratory laparotomy.

**Table 2:** Diagnostic modalities for IPMN derived from ectopic pancreas

	*Total* N *= 13*	*Stomach* n *= 6*	*Duodenum* n *= 1*	*Jejunum* n *= 3*	*Ileum* n *= 1*	*Meckel diverticulum* n *= 2*	P *value*
Diagnostic modality							.343
EGD	2 (15.4)	2 (33.3)	0 (0)	0 (0)	0 (0)	0 (0)	
Endoscopic ultrasound	1 (7.7)	1 (16.6)	0 (0)	0 (0)	0 (0)	0 (0)	
Ultrasound	1 (7.7)	1 (16.6)	0 (0)	0 (0)	0 (0)	0 (0)	
CT scan	3 (23.1)	1 (16.6)	1 (100.0)	0 (0)	1 (100.0)	0 (0)	
MRI	2 (15.4)	2 (33.3)	0 (0)	0 (0)	0 (0)	0 (0)	
Operation	6 (46.2)	1 (16.6)	0 (0)	3 (100.0)	0 (0)	2 (100)	
Exploratory laparotomy	3 (23.1)	0 (0)	0 (0)	1 (33.3)	0 (0)	2 (100)	
Incidental finding	3 (23.1)	1 (16.6)	0 (0)	2 (66.6)	0 (0)	0 (0)	

### Surgery and Pathologic Features of IPMN Derived From Ectopic Pancreas

All patients underwent surgery (Table 3). The tumor was detected preoperatively in 5 patients. Complications such as small bowel obstruction and perforation of the sigmoid colon were present in 2 patients and 1 patient, respectively. For tumors in the stomach, partial gastrectomy, including percutaneous endoscopic intragastric surgery, was performed in 4 patients; antrectomy in 1 patient; and pancreaticoduodenectomy for concomitant IPMC in the pancreas in 1 patient. Partial resections were performed for tumors in the small bowel. Concomitant pancreatic diseases consisted of IPMN in 1 patient, IPMC in 1 patient, and pancreatic cancer in 1 patient.

**Table 3:** Surgical and pathological characteristics for IPMN derived from ectopic pancreas

*Characteristics*	*Total* N *= 13*
Preoperative diagnosis	
Cystic tumor	5 (38.5)
Submucosal tumor	2 (15.4)
Small bowel obstruction	2 (15.4)
Perforation of the sigmoid colon	1 (7.7)
Operation performed (%)	13 (100.0)
Type of surgery (%)	
Antrectomy	1 (7.7)
Partial gastrectomy	3 (23.1)
Percutaneous endoscopic intragastric surgery	1 (7.7)
Laparoscopic duodenal partial resection	1 (7.7)
Pancreaticoduodenectomy	1 (7.7)
Small bowel resection	4 (30.8)
Small bowel resection and Hartmann's operation	1 (7.7)
Concomitant pancreatic disease (%)	3 (23.1)
IPMN	1 (7.7)
IPMC	1 (7.7)
Pancreatic cancer	1 (7.7)
Size of the tumor (cm) (range)	2.5 (0.7–6.0)
Histopathological diagnosis of the tumor at ectopic pancreas (%)	
IPMN	9 (69.2)
IPMA	1 (7.7)
IPMC	3 (23.1)
Histopathologic cell type (%)	
Gastric type	3 (23.1)
Intestinal type	1 (7.7)
Heinrich classification I/II/III	3/0/0
R0 resection (%)	13 (100.0)

*IPMA*, intraductal papillary mucinous adenoma.

Heinrich classification: type I, ducts plus acini plus endocrine islets; type II, ducts plus acini; and type III, ducts with a few acini or dilated ducts only.

The median size of the tumor was 2.5 cm (range, 0.7–6.0 cm). Histopathological examination of the tumor revealed IPMN in 9 patients, IPMC in 3 patients, and intraductal papillary mucinous adenoma in 1 patient. Histopathologic cell types included gastric type in 3 patients (the tumor was in the jejunum in 2 patients and stomach in 1 patient) and intestinal type in 1 patient. Heinrich type I ectopic pancreas was found in 3 patients only [19]. R0 resections were performed in all patients.

### Postoperative Treatment and Prognosis

No adjuvant treatment was provided for the tumor derived from the ectopic pancreas. However, adjuvant chemotherapy for concomitant pancreatic cancer was administered to the patient from our hospital. In this patient, newly recurrent liver lesions from pancreatic cancer were detected 4 months after surgery. Survival outcome data were available for 8 patients, including the patient from our hospital. The median observation period was 15 months (range, 1–72 months). Except for the above, no recurrence was found. The overall 5-year survival rate was 100%.

### Comparison of Characteristics Between IPMN and IPMC Derived From Ectopic Pancreas

Of the 13 patients, 3 patients had IPMC (Table 4), and 10 patients had IPMN. There were no differences in age (*P* = .932) or sex (*P* = 1) between the 2 groups. IPMC was detected only in the duodenum and jejunum. Six patients with IPMN had lesions in the stomach. IPMC was detected using CT in 1 patient, during exploratory laparotomy for perforation of the sigmoid colon in 1 patient, and incidentally during surgery in 1 patient. IPMC was diagnosed preoperatively only in 1 patient. The size of the tumor in the IPMC group was larger than that in the IPMN group, although the difference was not significant (*P* = .611).

**Table 4:** Comparison of characteristics between IPMN and IPMC derived from ectopic pancreas

*Characteristics*	*Total* N *= 13*	*IPMN n = 10*	*IPMC n = 3*	P *value*
Age (range)	69 (42–80)	67.5 (42–80)	69 (59–74)	.932
Sex (male/female)	10/3	8/2	2/1	1
Location of ectopic pancreas				.059
Stomach	6 (46.2)	6 (60.0)	0 (0)	
Duodenum	1 (7.7)	0 (0)	1 (33.3)	
Jejunum	3 (23.1)	1 (10.0)	2 (66.6)	
Ileum	1 (7.7)	1 (10.0)	0 (0)	
Meckel diverticulum	2 (15.4)	2 (20.0)	0 (0)	
Diagnostic modality				.811
EGD	2 (15.4)	2 (20.0)	0 (0)	
Endoscopic ultrasound	1 (7.7)	1 (10.0)	0 (0)	
Ultrasound	1 (7.7)	1 (10.0)	0 (0)	
CT scan	3 (23.1)	2 (20.0)	1 (33.3)	
MRI	2 (15.4)	2 (20.0)	0 (0)	
Operation	6 (46.2)	4 (40.0)	2 (66.6)	
Exploratory laparotomy	3 (23.1)	2 (20.0)	1 (33.3)	
Incidental finding	3 (23.1)	2 (20.0)	1 (33.3)	
Preoperative diagnosis				.867
Cystic tumor	5 (38.5)	4 (40.0)	1 (33.3)	
Submucosal tumor	2 (15.4)	1 (10.0)	0 (0)	
Small bowel obstruction	2 (15.4)	2 (20.0)	0 (0)	
Perforation of the sigmoid colon	1 (7.7)	0 (0)	1 (33.3)	
Operation performed (%)	13 (100.0)	10 (100.0)	3 (100.0)	1
Type of surgery (%)				.294
Antrectomy	1 (7.7)	1 (10.0)	0 (0)	
Partial gastrectomy	3 (23.1)	3 (30.0)	0 (0)	
Percutaneous endoscopic intragastric surgery	1 (7.7)	1 (10.0)	0 (0)	
Laparoscopic duodenal partial resection	1 (7.7)	0 (0)	1 (33.3)	
Pancreaticoduodenectomy	1 (7.7)	1 (10.0)	0 (0)	
Small bowel resection	5 (38.5)	4 (40.0)	1 (33.3)	
Small bowel resection and Hartmann's operation	1 (7.7)	0 (0)	1 (33.3)	
Concomitant pancreatic disease (%)	3 (23.1)	2 (20.0)	1 (33.3)	1
IPMN	1 (7.7)	1 (10.0)	0 (0)	
IPMC	1 (7.7)	1 (10.0)	0 (0)	
Pancreatic cancer	1 (7.7)	0 (0)	1 (33.3)	
Size of the tumor (cm) (range)	2.5 (0.7–6.0)	2.35 (0.7–6.0)	3.0 (1.8–5.0)	.611
Histopathologic cell type (%)				1
Gastric type	3 (23.1)	2 (20.0)	1 (33.3)	
Intestinal type	1 (7.7)	1 (10.0)	0 (0)	
Heinrich classification I/II/III	3/0/0	2/0/0	1/0/0	1

Heinrich classification: type I, ducts plus acini plus endocrine islets; type II, ducts plus acini; and type III, ducts with a few acini or dilated ducts only.

## DISCUSSION

We conducted a review and retrospective analysis of data of 13 patients with IPMN derived from the ectopic pancreas, with a focus on clinicopathological features and outcomes. Clinically, it is challenging to diagnose IPMN derived from the ectopic pancreas before surgery despite advances in modern imaging modalities. However, the number of cases of IPMN derived from the ectopic pancreas is expected to increase. Therefore, the clinicopathological features of the tumor should be clarified. This study is the first review of the literature regarding IPMN derived from the ectopic pancreas.

Of the 13 patients with IPMN derived from the ectopic pancreas, we found that 10 were men and 3 were women, and the median age was 69 years (range, 42–80 years), ie, it was more commonly observed in middle-aged to older men than in women. Pearson et al reported the frequencies of the ectopic pancreas as follows: 30% in the duodenum, 25% in the stomach, 15% in the jejunum, 3% in the ileum, and 6% in the Meckel diverticulum [20]. In the present study, the presence of IPMN was confirmed at these anatomic sites, and the stomach was the most common site. This is probably because the stomach is easily accessed by EGD. In fact, this condition was diagnosed by EGD in 2 patients.

In the present study, 5 patients had no symptoms. In 4 out of 5 patients, the tumors were incidentally found during routine examinations or evaluations for other conditions. It is not completely understood whether resection should be performed for these tumors. Adenocarcinoma, neuroendocrine tumors, and other cystic tumors in the ectopic pancreas have also been reported [21]. Thus, physicians should know that IPMN can emerge from the ectopic pancreas and that these tumors may have a malignant potential, although histological determination of carcinoma that has developed from pre-existing heterotopic tissue may be difficult because our results revealed that they were small with median size of 2.5 cm and none of 13 patients was diagnosed with malignancy preoperatively. Moreover, most small intestinal tumors are malignant, and nearly half of them are accounted for by metastatic malignant tumors [22,23]. Therefore, these tumors should be resected even if they are found incidentally.

**Fig 5:**
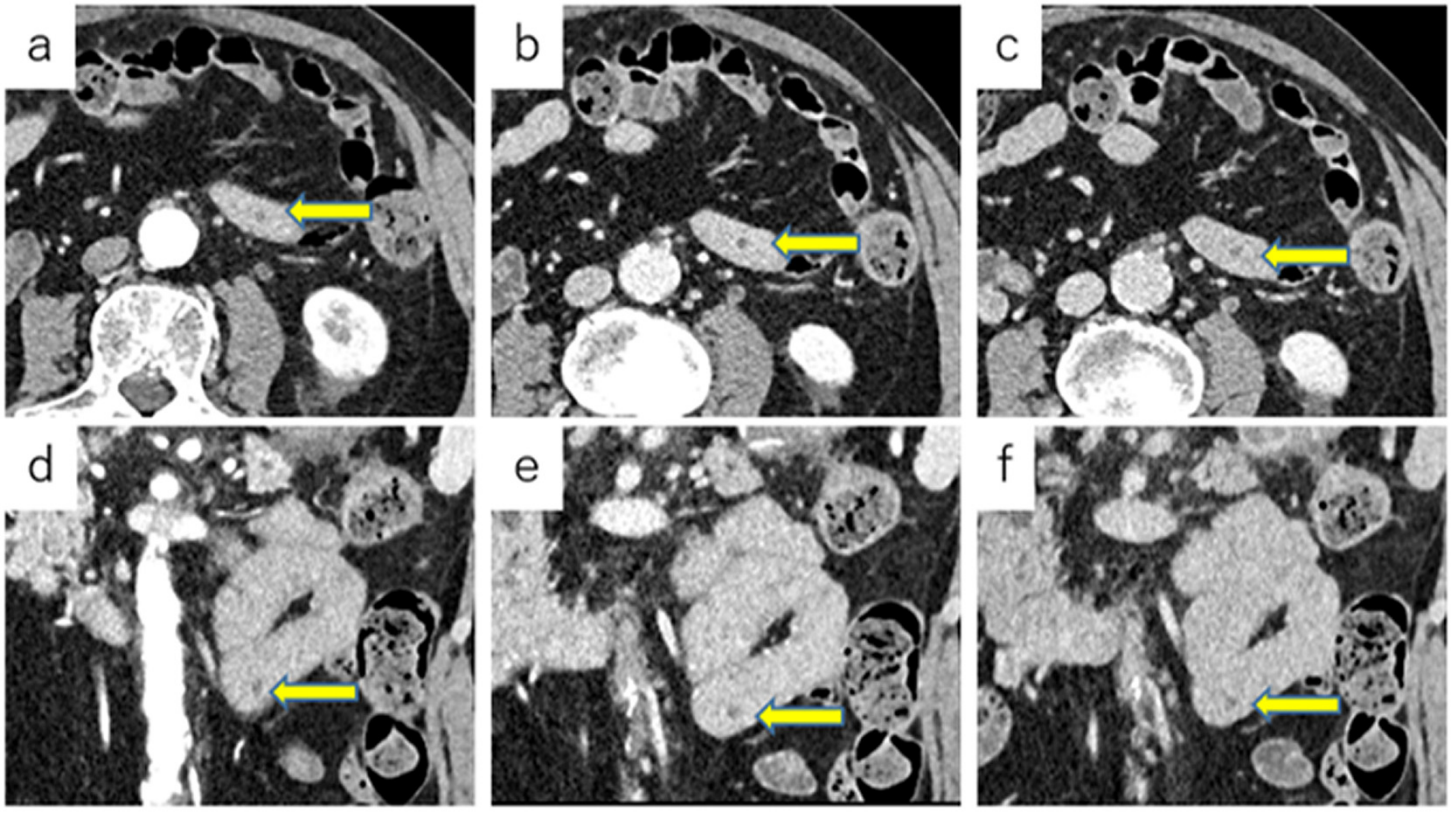
Contrast-enhanced CT scan revealed a homogeneous cystic lesion that was 5 mm in diameter without contrast (arrow). A, Axial view, arterial phase. B, Coronal view, arterial phase. C, Axial view, portal phase. D, Coronal view, portal phase. E, Axial view, equilibrium phase. F, Coronal view, equilibrium phase.

Cases of IPMN derived from ectopic pancreas have been reported since 2005. Despite advancements in imaging technology, some of these tumors are still diagnosed intraoperatively. In the present study, this condition was diagnosed intraoperatively in 6 patients. Of these patients, 2 patients with small bowel obstruction and 1 patient with perforation of the sigmoid colon underwent exploratory laparotomy. Tumors of the remaining 3 patients were incidentally found during surgery for abdominal aortic aneurysm, IPMN, and pancreatic cancer, respectively. In the case of the patient from our hospital, the tumor could not be detected using CT or MRI preoperatively; however, retrospective evaluation of the CT images barely enabled us to detect the cystic tumor with 5 mm in diameter (Fig 5). In contrast, MRI did not reveal the presence of the tumor even postoperatively because of its small size. Therefore, in some cases, it was difficult to diagnose the tumor preoperatively.

Considering the opportunity for organ-wise diagnosis, 3 patients had abdominal symptoms, and 5 patients were diagnosed with a lesion in the stomach preoperatively. Four lesions were suspected to be cystic tumors, and one was a submucosal tumor. In a patient with upper abdominal discomfort, a multicystic tumor in the duodenum was detected using CT. Similarly, in another patient, a follow-up CT for hereditary nonpolyposis colon cancer revealed a submucosal tumor in the ileum. However, all 5 patients were diagnosed with tumors in the jejunum and Meckel diverticulum intraoperatively. This was because of the difficulty in detecting relatively small lesions in the region from the jejunum to ileum and the rarity of such lesions.

Imaging modalities revealed the characteristics of the tumors. They were recognized as multicyclic lesions in 3 patients, submucosal masses in 2 patients, subepithelial cyst in 1 patient, and unilocular cyst in 1 patient. Therefore, when a submucosal cystic lesion occurs in the gastrointestinal tract, IPMN derived from the ectopic pancreas should be considered as a differential diagnosis.

The difference between IPMN and IPMC derived from ectopic pancreas was investigated in this study. No significant differences were observed. No malignant lesions were observed in the stomach. Moreover, IPMC was detected only in the duodenum and jejunum. This might indicate that small bowel lesions were difficult to detect because enteroscopy could not be performed at any of the institutions or was not performed on a regular basis. In fact, enteroscopy was not performed for any patient in the present study. Further, lesions in the stomach might have been detected earlier. In fact, in 2 patients, lesions in the stomach were detected by EGD. Only 1 patient with IPMC was diagnosed with a cystic lesion preoperatively. It was a multicystic lesion without nodular formation with a diameter of 5 cm. However, it was difficult to precisely diagnose IPMC. Another patient with IPMC had a perforation of the sigmoid colon due to invasion of the IPMC lesion, with a diameter of 3 cm, located in the jejunum. In general, the size of the tumor in patients with IPMC was larger than that in patients with IPMN, although the present study did not reveal a significant difference in size (*P* = .611). Thus, it is difficult to define risk factors for IPMC.

The present study has some limitations associated with the errors and biases inherent to a retrospective study with small sample size. A major limitation of this study was that the results could not be estimated simply because the study was a review of previously published case reports. Another limitation was that the information obtained from each case report was limited and biased and had missing values. Therefore, collection and analysis of further epidemiological and pathological data are necessary to obtain more accurate results and determine the best management strategy for IPMN/IPMC derived from the ectopic pancreas.

In conclusion, although some patients with IPMN derived from the ectopic pancreas are asymptomatic and the condition may remain undiagnosed until the lesion is incidentally found, malignant transformations can occur. Accurate preoperative diagnosis and differential diagnosis between IPMN and IPMC remain difficult despite recent advances in imaging modalities. Further analysis of risk factors for malignant transformation to IPMC is essential.

## Author Contribution

TO contributed design of the work. KS, TM, MT, and MA research data. JI analyzed data. JK and TO wrote the manuscript and researched data. YH reviewed/edited the manuscript. JI, TO, and JI contributed to discussion and reviewed/edited the manuscript.

## Conflict of Interest

The authors declare that they have no competing interests.

## Funding Source

This work was supported by Kochi Organization for Medical Reformation and Renewal grants, and the inochino-kikin aided THE KOCHI SHIMBUN and Kochi Broadcasting Co, Ltd (2020–4).

## Ethics Statement

This study was approved by the Ethics Committee of Kochi Health Sciences Center.
